# *Galleria mellonella* as a screening tool to study virulence factors of *Aspergillus fumigatus*

**DOI:** 10.1080/21505594.2021.1893945

**Published:** 2021-03-08

**Authors:** Marie-Fleur Durieux, Élise Melloul, Sana Jemel, Lolita Roisin, Marie-Laure Dardé, Jacques Guillot, Éric Dannaoui, Françoise Botterel

**Affiliations:** a Laboratoire de Parasitologie - Mycologie, CHU de Limoges, Limoges, France; b EA 7380 Dynamic, Université Paris Est Créteil, EnvA, USC ANSES, Créteil, France; c École Nationale Vétérinaire d’Alfort, Maisons-Alfort, France; d Unité de Parasitologie-mycologie, Service de Microbiologie, Université Paris Descartes, Hôpital Européen Georges Pompidou, AP-HP, Paris, France; e Unité de Mycologie, Département de Prévention, Diagnostic Et Traitement Des Infections, Groupe Hospitalier Henri Mondor - Albert Chenevier, APHP, France

**Keywords:** Review, *Galleria mellonella*, *aspergillus fumigatus*, mini-host model, virulence factors

## Abstract

The invertebrate *Galleria mellonella* has increasingly and widely been used in the last few years to study complex host–microbe interactions. *Aspergillus fumigatus* is one of the most pathogenic fungi causing life-threatening diseases in humans and animals. *Galleria mellonella* larvae has been proven as a reliable model for the analysis of pathogenesis and virulence factors, enable to screen a large number of *A. fumigatus* strains. This review describes the different uses of *G. mellonella* to study *A. fumigatus* and provides a comparison of the different protocols to trace fungal pathogenicity. The review also includes a summary of the diverse mutants tested in *G. mellonella*, and their respective contribution to *A. fumigatus* virulence. Previous investigations indicated that *G. mellonella* should be considered as an interesting tool even though a mammalian model may be required to complete and verify initial data.

## Introduction

Rodent models are the gold standard in clinical studies and *in vivo* experiments and have been extensively used for a better understanding of the physiopathology of infectious diseases. International fundamental regulation 3Rs rules (Replacement, Reduction, and Refinement) guarantee welfare of animals and encourage researchers to replace traditional rodent models with alternative, non-mammalian models [1]. Since the early 2000s, and particularly over the last few years, many articles on invertebrate and mini-host models have been published in the literature. Until now, the ethical rules have never been applied to the use of insects and nematodes [2]. For instance, fruit fly *Drosophila melanogaster* has been the best-known invertebrate model used in genetic and developmental biology studies for over 100 years [3]. Other invertebrates such as the beetle *Tribolium castaneum*, the nematode *Caenorhabditis elegans*, the butterfly *Bombyx mori*, the moth *Galleria mellonella*, or the non-mammalian vertebrate model *Danio rerio* are also used [3–7]. Their genome, immunity, and physiology were analyzed through many environmental and medical studies. In microbiology, *C. elegans, D. melanogaster*, and *G. mellonella* have recently been demonstrated as interesting tools to evaluate the virulence and the pathogenesis of human pathogens, e.g. fungi. These models were successfully used in virulence assays, immunity tests, histopathology analyses, or new antimicrobial drugs testings [8].

**Figure 1.**
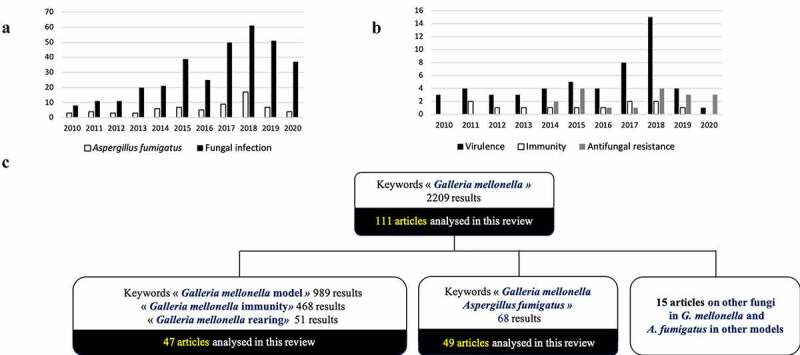
Publications mentioning *A. fumigatus* and *G. mellonella* on Pubmed

One of these alternative models, *G. mellonella*, has attracted increasing attention in recent years because of the many advantages it provides to study microorganisms. *Galleria mellonella* is become one of the most popular invertebrate models with more than 2,200 scientific articles published (search terms “*Galleria mellonella”* on Pubmed) (Figure 1). The moth *G. mellonella* belongs to the Lepidoptera order and is present worldwide as a ubiquitous pest of honeybees that destroys honeycombs by feeding on bee wax, honey, and bee pollen [9]. In research laboratories, its last larval stage can be used, just before transformation into a pupa. Many recent reviews [10–15] describe very well all the advantages and disadvantages of this mini-host model, and some benefits deserve attention. Larvae are naturally exposed to pathogens and have developed immune defense systems, which have many similarities with the innate immune system of vertebrates. The moth innate immune system, mediated by hemocytes, can fight against a large spectrum of pathogens via phagocytosis, melanization, and secretion of antimicrobial peptides [16]. Other intriguing points are its fast and high reproductive rate at low cost and the easy maintenance of its larvae in laboratory without the need for expensive equipment [17,18]. In comparison with other invertebrate models, *G. mellonella* can survive within a wide temperature range (18°C to 37°C) [3,5], an essential point to mimic mammals physiology and facilitate the study of human pathogens. Furthermore, the genome of *G. mellonella* was entirely sequenced in 2018 [19], which makes it easy to have well-defined populations of larvae, and perhaps allows to create a biobank with database as with other invertebrate models, Flybase and WormBase [12]. In our experience and according to Amorim-Vaz *et al*., Eisenman *et al*., and more recently Champion *et al*., the main limitation of this model is the difficulty to have reproducibility of results compared with the mice models [20–22]. The reasons for this are probably the origin of larvae, the different rearing conditions, temperature of storage, nutrition, genetics, and age of larvae used in the experiments [23–26]. This limitation highlights the need for standardization to make *G. mellonella* a more reliable model [22].

(A) Evolution of the number of publications on *A.fumigatus* and *G.mellonella* over the period 2010 – august 2020; (B) Details of the publication on *A. fumigatus* studies with virulence, resistance to antifungal in *G. mellonella*; (C) number of articles in the literature and in the review on the *G. mellonella* model

*Galleria mellonella* has been used for the complex study of host–microbe interactions, especially host-fungi interactions [4,6,24–30]. This model is now recognized as a pertinent model to the study of the fungal infections [31,32]. *Aspergillus fumigatus* remains one of the most common pathogenic fungi known to colonize the respiratory tract of patients with chronic lung diseases (e.g. cystic fibrosis), and to cause invasive fungal infections in immunocompromised patients [33–35]. The mini-host model *G. mellonella* has been used to evaluate the virulence of *A. fumigatus*, where mutants are tested to investigate the role of specific protein in the pathogenicity, and eventually to try to find a target for novel antifungal therapies.

This review aims to compare the different protocols published in the literature to study *A. fumigatus* in *G. mellonella*, and to present the virulence studies already conducted in this mini-host model. We also present the currently available literature concerning the virulence of *A. fumigatus* in a *G. mellonella* model with several clinical and environmental strains including data obtained in our research team [29]. This study does not tackle the antifungal treatments tested on *A. fumigatus* in the *G. mellonella* model. The latter are included in an additional review of our team and in other recent articles [36,37]

## *Galleria mellonella* model

**Figure 2.**
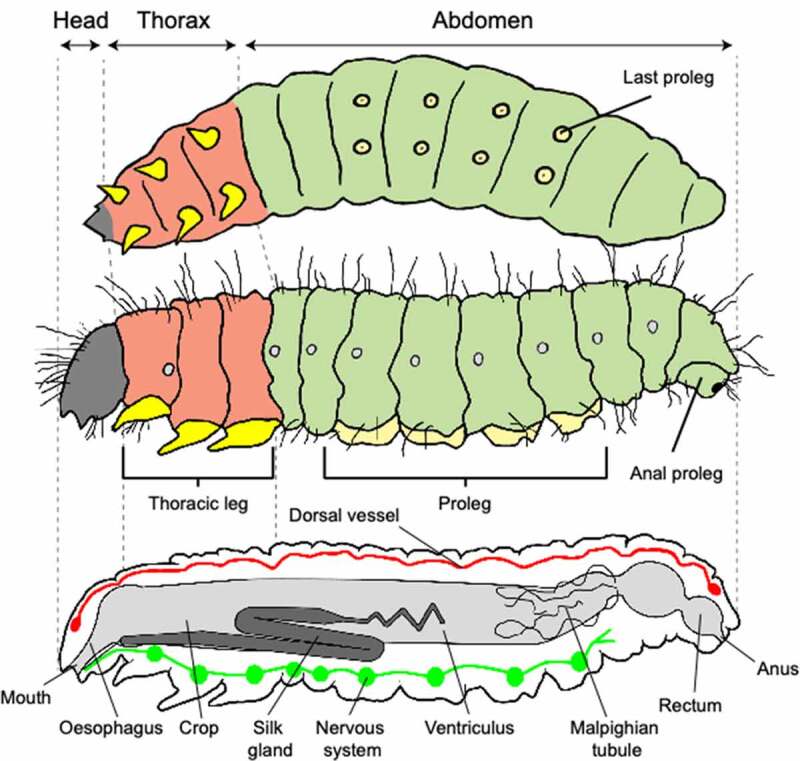
Anatomy of a larva of *Galleria mellonella*, adapted from Singkum *et al*., 2018 [15] and Engel and Moran, 2013 [40]

*Galleria mellonella*, also known as the wax moth, belongs to the *Pyralidae* family in the Lepidopteran order. Morphology and characteristics of every stage of its lifecycle are precisely described in Kwadha *et al*. and Ellis *et al*. [9,17]. Briefly, the larvae have six legs on the thorax, eight prolegs on the abdominal segment, a digestive tube, vessels, silk glands, and a nervous system (Figure 2). Duration of its life cycle can vary from weeks to month depending on several factors, especially food, temperature, and humidity. An artificial food composed of a blend of honey and various cereals [38,39] can be used, but the need for food diminishes with the successive metamorphosis. Thus, food composition could have an impact on pupation, larval stage duration, volume of hemolymph, and density of hemocytes. Larvae can spin a silken thread in all stages, but they surround themselves with a cocoon only during their last stage. Now it is well known that food and environmental condition, such as temperature, humidity and darkness could play a role in the susceptibility to infection. That is why the scientific community endeavors to uniform breeding procedure to limit this source of bias. Jorjao *et al*. and more recently Firacative *et al*. proposed an optimal method to rear *G. mellonella* in laboratories for microbiological studies (dietary components, description of environmental conditions, and a detailed protocol for all life stages of *G. mellonella*) [32,38].

### *Immune response of* G. mellonella to A. fumigatus

In insects, only the innate immune system is effective and can protect against a large spectrum of pathogens including fungi [41,42]. *G. mellonella* immune system is an open circulating system of which hemolymph is the key element. This innate immune system comprises three parts: (i) physical barrier, (ii) cellular and (iii) humoral immune systems [13]. The cuticle, composed of chitin and many proteins with antimicrobial properties, represents the first protection line, that acts as a barrier to prevent the entry of pathogens. The cellular component consists of several types of cells, called hemocytes [16,43], circulating in the hemolymph to ensure of phagocytosis [44,45], encapsulation, and clotting activities [13]. At early stages of infection, the increase in circulating hemocyte density is due to release of attached hemocytes from internal organs. Furthermore, the humoral component, released by the hemolymph and body fat, consists of soluble effector molecules including opsonin, e.g. ApoLp-III, a pattern recognition molecule which can bind to ß-1,3 glucan of numerous fungus cell wall [46]. Another element of the humoral system is lytic enzymes which harbor several antimicrobial peptides (AMPs).

**Figure 3.**
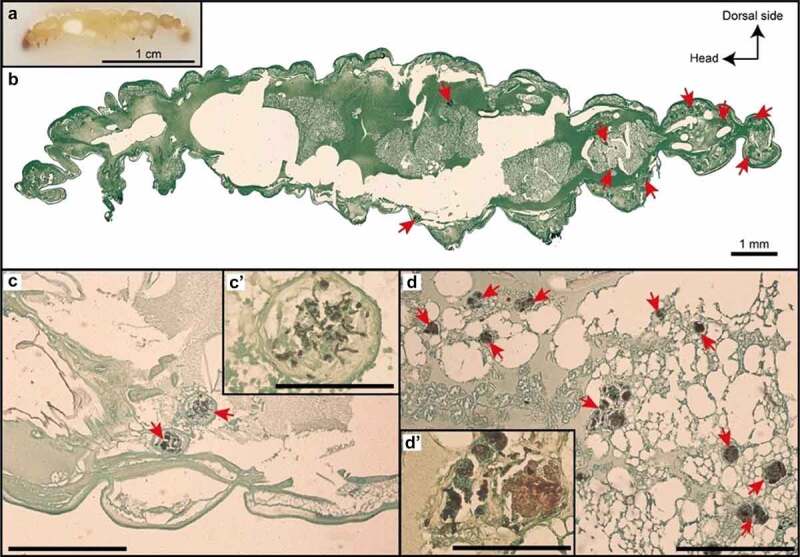
Histological analysis of *A. fumigatus* infection in *Galleria mellonella.*

The process of melanization, a fundamental role of the humoral system in arthropods, is activated upon the penetration of a foreign particle into the larva body [13]. Melanin synthesis, catalyzed by phenoloxidase, limits the spread of microorganisms through the formation of nodules visible on histological sections. Once inside the larva, *A. fumigatus* stimulate hemocytes to increase in density at early stages of infection (2 h). However, the action of several fungal toxins, such as fumagillin and gliotoxin, can counterbalance the physical action of the fungus by inhibiting the action of hemocytes [47,48]. After several hours, *A. fumigatus* can invade larvae with hyphae. This process results in the formation of nodules disseminated all over the body of larva and not only near the site of inoculation (Figure 3). If the infection is controlled by the immune system, the larva can survive. On the contrary, if the immune system cannot control infection, the larvae become completely melanized and die. Many factors can influence the immune response of larvae, such as physical, nutritional, thermal stress, or exposure to cell wall components [44–49]. Recently, Sheehan *et al* described the immune response of *G. mellonella* larvae and the factors influencing it [49].

Sagittal section of *G. mellonella* infected with *A. fumigatus*. (A) Larvae fixed in paraffin; (B) Gomori-Grocott stain; (C) *A. fumigatus* nodules after 3 days of infection (10^5^ conidia/larva); (D) *A. fumigatus* nodules after 7 days of infection (10^6^ conidia/larva)

## Experimental design for virulence assay

### Infection with filamentous fungi

Homogenous groups of 10 to 20 larvae with cream-color cuticle, of about 200–300 mg weight, 1–3 cm length and spontaneous mobility, are generally used. The larvae should be manipulated delicately to avoid physical stress. After infection, larvae are maintained up to 37°C without feeding. Control groups are also essential to ensure that the trauma of inoculation or the use of buffer do not affect the larvae survival. Three methods are used to infect the larvae: topical application, ingestion, and injection.

Topical application of fungi causes penetration into the exoskeleton, which is close to natural fungal contamination. Some authors utilized this trauma-free method to inoculate larvae by immersion in a conidial suspension of *Beauveria bassiana* for about 10s [50,51]. In another study, 5 µL containing *Aspergillus flavus* (1x10^3^ to 1 × 10^8^ conidia/mL) was directly applied on the dorsal surface of larvae [52]. Overall, this method is rarely used because of reproducibility issues linked to the difficulty having a precise inoculum delivery into the larvae.

Forced-feeding (ingestion) consists in inserting 10 µL of fungal suspension into the larval mouthpart using a micro-injector and a needle [53,54,68].

The preferred and the most commonly used method to study interaction between filamentous fungi and *G. mellonella* is the injection of inoculum into larval hemocoel by pricking the cuticle with a needle at the last proleg [26]. The last left proleg is the preferred site for injection but other sites are possible, if necessary [27]. The main upside of this method is the better delivery of a precise inoculum (5 to 20 µL per larva) using either an insulin or a Hamilton syringe. The latter is more precise to inoculate small volumes. An insulin syringe with an automatic applicator could be used for larger quantities and faster inoculations [26,55,56]. The injection is almost scarless for the larva but there is some risk for the operator (especially for BSL-3 microorganisms) that could be avoided with proper restraint and handling techniques [55,57]. Differences between the employed protocols in terms of inoculum preparation, technique of inoculation, and experimental conditions are described in Table 1.

**Table 1.** Comparison between protocols to analyze virulence of *A. fumigatus* in *G. mellonella.*

Ref.	Larva selection criteria	Larva/group	Inoculation (in hemocoel)	Inoculum conidia/larva	Maintenance of larva		Monitoring (during of experiment)
					Before inoculation	After inoculation	
[85,86,96]	0.3–0.5 g No gray marking	16	10 µL	5x10^5^	/	In Petri dishes, in the dark, at 37°C	Every 12 h (8 days)
[95]	/	20	5 µL	5x10^6^	/	At 37°C	Daily (5 days)
[88,91,94]	6th -instar larvae	15	5 µL	2x10^5^	/	At 37°C	Daily (5 days)
[43]	6^th^ -instar larvae 0.2–0.4 g	20	20 µL Myjector U-100 Insulin needle	1x10^4^	In wood shavings, in the dark, at 15°C	In Petri dishes, in the dark, at 30°C	Daily (5 days)
[29,58]	/	10	10 μL Hamilton syringe	∼ 3 × 10^6^ to 3 × 10^3^	/	At 37°C	Daily (7 days)
[108]	/	30	20 µL	1x10^6^ or 1 × 10^7^	/	/	Daily (7 days)
[59,60,112]	6^th^ -instar larvae 0.3–0.4 g	20	20 µL	1x10^7^	In the dark, at 18°C	In the dark, at 30°C	Daily (6 days)
[110]	0.275 and 0.300 g No gray marking	30	Hamilton syringe	1x10^6^	/	In Petri dish, in the dark, at 37°C	After 16 h, every 2 h (30 h)
[61]	6^th^ -instar larvae	/	20 µL	1x10^7^	In the dark, at 18°C	In the dark, at 30°C	Daily (6 days)
[106]	6^th^ -instar larvae 0.2–0.4 g	/	Myjector U-100 insulin syringe	1x10^4^ to 1 × 10^7^	In the dark, at 15°C	At 30°C	/
[97]	6^th^ -instar larvae 0.25–0.35 g	12	5 µL Hamilton syringe	5x10^5^	/	In Petri dishes, in the dark, at 37°C	Daily, (5 days)
[62]	/	30	5 µL	5x10^7^	/	/	Every 12 h (5 days)
[109]	No gray marking 0.2 g	10 to 15	20 μL Disposable 29.5-gauge hypodermic needle	5x10^5^ 2x10^5^	/	/	Daily (7 days)
[63]	Final-instar larvae 0.2 g	10	10 µL Hamilton syringe	1x10^5^	In wood shavings, in the dark	In Petri dishes, in the dark, at 37°C	Daily (5 days)
[64]	Final-instar larvae 0.275–0.300 g	10	5 µL	1x10^6^	Without food, at 37°C, in the dark for 24 h	In Petri dishes, in the dark, at 37°C	Daily (10 days)
[76]	Sixth instar larvae	/		1x10^5^	/	In the dark, at 37°C	Daily (10 days)
[77]	6^th^ -instar larvae 0.3 g	/	5 µL	1x10^5^	/	In the dark, at 37°C	Daily (10 days)
[65]	6^th^ -instar larvae	30 to 35	5 μL Hamilton syringe	5x10^6^	In wood shavings, in the dark, at room temperature	In Petri dishes, in a dark humidified incubator at 37°	Daily (8 days)
[89]	0.30–0.35 g	16	10 µL	5x10^5^	/	In the dark, at 37°C	Every 12 h (5 days)
[66]	/	30	20 μL	5x10^6^	/	/	Daily (3 days)
[97]	0.275–0.300 g	30	10 µL	1x 10^5^ or 1x10^6^	/	In Petri dishes, in the dark, at 37°C	After 16 h, every 2 h (7 days)
[107]	6^th^-instar larvae 0.2–0.4 g	20 or 30	20 µL	1x10^6^ or 1x10^7^	In wood shavings, in the dark, at 15°C	/	Daily (4 days)
[67)	/	12–28	20 µL	8x10^4^	/	At 37°C	Daily (7 days)
[98]	0.3–0.5 g	10	10 µL	5x10^5^	At 8°C	In the dark, at 37°C	Every 8 h (6 days)
[90]	0.275–0.300 g	10	5 µL	1x10^6^ (37°C) 5x10^6^ (30°C)	/	At 37°C or 30°C	Every 12 h (6 days)
[81]	/	30	10 µL Hamilton 1 mL gas-tight syringe	1x10^8^ to 1x10^3^	In wood shavings, in the dark, at 4°C for up to 10 days	In Petri dishes, in the dark, at 37°C	Daily (7 days)
[83]	0.25–0.30 g	10	5 μL Hamilton syringe 25 μL	500 CFU/μL	/	In Petri dishes, in the dark, at 37°C, with pine wood chips	Daily (8 days)
[69]	6^th^ instar larvae 15–25 mm length	30	10 μL Braun Omnican 50-U 100 0.5 mL insulin syringe	1x10^6^	/	In the dark, at 37°C	Daily (7 days)

### Follow-up of *A. fumigatus* infection

#### Mortality monitoring

For the analysis of strains virulence, larvae survival is monitored over time after inoculation, most often every 24 h for 5 days. Larvae movements gradually decreases, reflecting the progression of fungal infection, but to a variable extent depending on fungal species and strains. Determining the best concentration of fungal inoculum is crucial to achieve a substantial killing rate [27]. Inoculum-finding experiments allow to calculate the median and 90% lethal doses (LD50 and LD90), and to compare the survival after wild-type and mutant strains inoculation to assess different virulence factors [56,71].

#### Morbidity monitoring

Another method can be used to assess the morbidity of larvae, based on several criteria of follow-up. The evaluation of morbidity gives more details on the progression of infection within the larvae. A scoring system that comprises four main criteria, melanization, mobility, capacity to form silk cocoon, and survival, has been used in some studies [14,22,72]. Melanization is an immune process visible to the naked eye and deemed completed when the larva is dead, and the immune response is overtaken. Thus, the degree of melanization is correlated with morbidity and a key element to assess the general condition of the larva. Larval mobility is evaluated individually on spontaneous and stimulated movements. The capacity of larva to turn around and move forward is a strong indicator of good health. The same is said for its capacity to form a silk cocoon. Initially, when the larva is not infected, a whole, highly resistant cocoon forms around it. As the infection spreads, the ability of the larva to form a cocoon decrease. In the pre-mortem phase, the larva can only form a few silk threads. The last criterion is the larval survival as shown in the details of morbidity score and modified by our team (Table 2). Each group obtains a final score 24 h after injection that seems predictive of the end of the experiment [29].

**Table 2.** Examples of scores for monitoring pathogenicity in *Galleria mellonella.*

	**Loh *et al*., health index scoring system [**73**]**		**Melloul *et al*., 2018 [**29**]**	
**Category**	**Description**	**Score**	**Description**	**Score**
**Activity**	No movement Minimal movement on stimulation Move when stimulated Move without stimulation	0 1 2 3	No movement No turn around and minimal movement on stimulation Difficult turn around and weak spontaneous mobility Normal, able to turn around and move	0 1 2 3
**Cocoon formation**	No cocoon Partial cocoon Full cocoon	0 0.5 1	No cocoon Full cocoon	0 1
**Melanization**	Black larvae Black spots on brown larvae ≥3 spots on beige larvae <3 spots on beige larvae No melanization	0 1 2 3 4	Melanized larvae No melanized larva	0 1
**Survival**	Dead Alive	0 2	Dead Alive	0 1

#### Histological analyses

To study pathogenesis and the host–pathogen interactions, histological analysis is recommended, especially to describe tissue damage caused by fungal infection. A procedure was developed to analyze *C. albica*ns virulence and to assess morphological changes in larva body [73]. This procedure can also be used with other fungal species. It consists in injecting formalin into the larvae which are then stored at 4°C for a few days. Later, larvae are carefully dissected from sagittal or transversal lines and stained with Gomori-Grocott or Hematoxylin and Eosin (HE). Recently, Sheehan *et al*. have provided histological data on invasive *A. fumigatus* infection, and highlighted the usefulness of *G. mellonella* larvae, albeit they have no respiratory system [74]. Indeed, the development of invasive aspergillosis in larvae shows similarities to that occurring in mammals. Sheehan *et al*. showed that the inoculation of conidia is followed by (i) the formation of melanized nodules and (ii) an increase in the density of hemocytes and antimicrobial peptides. These nodules have a histological structure similar to the granulomas detected in the mouse model of aspergillosis. In their work, they utilized a technique that does not require the use of formalin: larvae were embedded in Bioinvision Cryo-Imaging Embedding Compound, and flash-frozen in liquid nitrogen. Then, slides were made with Cryoviz for a specific cryo-imaging.

Infected larvae melanized with time by forming melanized capsules that surrounded the pathogens, and their internal organs were disorganized by the infection. Many authors used the melanization or tissue damage to better understand the progress of infection and the effect of fungal mutants on larvae as visualized by histological analysis and Gomori-Grocott staining [75,76]. In a work of our team, we analyzed the progress of *A. fumigatus* infection in larvae after 3 days (A) and 7 days (B) with appearance of melanized nodules and granulomas containing both conidia and hyphae (Figure 3). Number and size of these granulomas, which are distributed all over the larva, increased over time after infection (personal data).

## *Galleria mellonella*-based screening to study virulence factors of *Aspergillus fumigatus*

### Origin of strains

The relationship between virulence and the origin of the strains has insufficiently been studied.

In rodents models, studies showed that *A. fumigatus* environmental isolates were less virulent than clinical isolates [77–79]. Similarly, Alshareef *et al*. observed that clinical strains (n = 10) appeared to be more virulent than the environmental ones (n = 20) in a *G. mellonella* model [80]. However, high variability was also observed between isolates of the same origin [80], even between isogenic strains isolated from a single chronic granulomatous disease patient [81].

Other studies showed opposite results; Cheema and Christians [82] observed a lower survival rate of *G. mellonella* larvae inoculated with environmental strains (n = 8) compared with clinical isolates (n = 8). In the same way, Knox *et al*. showed that two *A. fumigatus* isolates collected in the International Space Station were more lethal than the clinical reference strain in zebrafish model [83].

These discordant results preclude any conclusion of isolate origin effect on virulence of *A. fumigatus* in *G. mellonella* model. Moreover, a recent study [84] has analyzed the whole-genome sequence of *A. fumigatus* isolates to determine their virulence genes content and revealed a high genetic diversity between environmental and clinical isolates, as well as between clinical isolates from the same patient, but a similar virulence genes content.

Up to now, no animal-origin strains have been tested in *G. mellonella* model. In our team, we have tested for the first-time the pathogenicity of two different animal *A. fumigatus* strains collected from wild fauna (AF_A1) and from a duck (AF_A2) [29]. Ten larvae were infected by injecting the hemocoel with 10 µL at the concentration of 10^6^ conidia/larva. After 7 days of infection, AF_A1 had a 10% survival rate compared with 30% survival rate for AF_A2. The variability of virulence observed for the animal strains is similarly for the clinical and the environmental strains. These results are consistent with those of other studies. However, currently, no link could be established between the origin and the pathogenicity of *A. fumigatus* strains [80,82,84].

The relationship between virulence and fungal development (conidiation, germination, and fungal growth) involves several mechanisms not completely elucidated. Understanding these mechanisms is essential mainly to find new therapeutic targets against *A. fumigatus*. A large number of *A. fumigatus* mutants involved in these signaling pathways have been tested in *G. mellonella* model with sometimes discordant results, especially compared to mice models [85–98] (Table 3).

**Table 3.** List of *A. fumigatus* mutants tested in *Galleria mellonella.*

Function			Mutants	Reference *A. fumigatus* strain	*In vitro* effects on development, stress response, and metabolism	Virulence in *G. mellonella*	Virulence in mice	Ref.
	**SECONDARY METABOLITES**							
***mtfA*** gene encodes a putative C2H2 zinc finger domain-type transcription factor (fungal development and secondary metabolism)			***∆mtfA***	Af CEA10	Role in growth rate & gliotoxin production	Hypovirulent	**/**	[110]
			***OEmtfA***		Role in growth rate & conidiation, stress response, gliotoxin production	Normal	**/**	
***rtfA*** gene encodes a RNA polymerase II transcription elongation factor-like protein			***∆rtfA***	Af CEA17	Reduce growth rate, increase conidiation, oxidative stress, and metabolites metabolism	Hypovirulent	Normal (40)	[97]
			***rtfA OE***		No difference in growth, conidiation, stress response, or metabolites metabolism. Minor effect on cell wall.	Normal	/	
Gene cluster involved in melanin pathway		*Alb1*: encodes polyketide synthase	**Color mutant alb1**	Af 293	/	Hypervirulent	Hypovirulent	[100]
		*Ayg1*: encodes heptaketide hydrolyase	**Color mutant ayg1**			Hypervirulent	/	
		*Arp2*: encodes hydroxynaphthalene reductases	**Color mutant arp2**			Hypervirulent	/	
		*Arp1*: encodes scytalone dehydratases	**Color mutant arp1**			Hypervirulent	Hypovirulent	
		*Abr1*: encodes multicopper oxidase	**Color mutant abr1**			Hypervirulent	**/**	
		*Abr2*: encodes laccase	**Color mutant abr2**			Hypervirulent	**/**	
***dmaW, easM, A, G*** genes involved in ergot alkaloid pathway			**∆dmaW**	Af FGSC A1141	/	Hypovirulent	**/**	[109]
			**∆easM**	Af293		Hypovirulent	**/**	
			**∆easA/G**			Hypovirulent	**/**	
***Pes1, PesL***: nonribosomal peptide (NRP) synthetases involved in fumigaclavine C biosynthesis			**∆pesL**	∆akuB mutant background	Reduce tolerance to H_2_O_2_ but increase tolerance to menadione, essential for fumigaclavine C biosynthesis	Hypovirulent	/	[107]
			**∆pes1**	in ∆akuB mutant and ATCC 46,645 backgrounds		Normal	**/**	
			**∆pes1**	Af293.1	Alter conidial morphology & hydrophobicity. More susceptible to oxidative stress	Hypovirulent	**/**	[108]
	**DEVELOPMENT**							
***Rgs (RgsC, RgsD & GprK*)**: regulator of G protein signaling, crucial roles in upstream regulation of vegetative growth, development, secondary metabolism, and virulence			***∆rgsD***	Af293	Increase conidiation, stress response, gliotoxin and melanin production	Hypervirulent	**/**	[87]
			***∆rgsC***	Af293.6	Increase germination, reduce conidiation, growth, tolerance to H_2_O_2_ and gliotoxin production, modify cell wall	Hypovirulent	**/**	[76]
			***∆gprK***	Af293.1	Increase germination & reduce conidiation, tolerance to H_2_O_2_ and gliotoxin production	Normal	**/**	[77]
***NosA***: number of sexual spores, transcription factor			***∆nosA***	Af CEA17 ∆akuB	Slightly reduce conidiation and increase radial growth & germination	Hypervirulent		(115)
**Cofilin**: actin depolymerizing factor, role in actin cytoskeleton dynamic			**cofilin_teton_/cofilin ^S5E^**	Af CEA17 ∆ku80	Impair growth rate, regulate cell wall & modify resistance to H_2_O_2_	Hypovirulent	Normal	[86]
			***Cofilin OE***		Role in growth rate & cell wall, increase resistance to H_2_O_2_	Normal	Normal	[85]
			***D19A R21A***		Increase production of ROS, apoptosis and ergosterol levels	Hypervirulent	**/**	[89]
			***K36A***		Increase production of ROS, apoptosis and ergosterol levels	Hypervirulent	/	
**Myosin**: cytoskeleton component, member of actin-based motor proteins family			***∆myoB*** (class II)	Af akuBKU80 pyrG^−^	Delayed germination, increase of conidiation & modification of cell wall	Hypovirulent	Hypovirulent	[95]
			***∆myoE*** (class V)		Delayed germination, reduction of growth rate & conidiation, modification of cell wall	Hypovirulent	Normal	
			**∆myoB∆myoE**	*∆myoB*		/	/	
**Septins**: GTPases family, regulates cellular processes (cell wall integrity, septation)			**∆aspA**	Af *akuB^KU80^*	No difference in growth rate, reduce conidiation	Hypervirulent	/	[94]
			**∆aspB**		Reduce growth rate, reduce conidiation	Hypervirulent	Normal	
			**∆aspC**		No difference in growth rate, reduce conidiation	Hypervirulent	/	
			**∆aspD**			Normal	/	
			**∆aspE**			Normal	/	
			**∆aspE∆aspD**			Normal	/	
			**∆aspE∆aspB**			Hypervirulent	/	
			**∆aspE∆aspD∆aspB**			Hypervirulent	/	
***Rho1***: small GTPase, a potential regulatory subunit of β-1,3-glucan synthase			***Rho1_teton_***	Af CEA17Δku80	No growth & increase H_2_O_2_	Avirulent	**/**	[96]
			***Rho1 OE***		No difference in growth rate or in stress response	Normal	Normal	
***Kin1***: protein kinase is a member of the eukaryotic PAR-1/MARK/MELK family			***∆kin1***	Af akuB^KU80^	No difference in growth rate, conidiation & cell wall. Role in stress response	Normal	**/**	[91]
***srgA***: (secretion related GTPase A) encoding a Rab GTPase of the Rab family, master regulators of membrane trafficking			***∆srgA A***	AfS28	Reduce growth rate & aberrant conidiation, role in stress response	Normal	**/**	[97]
			***∆srgA B***			Normal	/	
			***∆srgA C***			Hypovirulent	/	
**ERMES**: Endoplasmic-Reticulum mitochondria encounter structure, tether between mitochondria and endoplasmic reticulum			***mmm1_tetOn_***	AfS35	Reduce growth rate	Hypovirulent	**/**	[98]
**Calcineurine**		***swoH***: nucleoside iphosphate kinase, interaction with the catalytic subunit of calcineurin (CalA)	**SwoH^V83F^**	Af CEA17-80	Reduce growth rate and increased sensitivity to elevated temperatures	Hypovirulent	**/**	[85]
		***cnaA***: calcineurin A (cnaA) catalytic subunit	***∆cnaA***	Af293.1	Reduce germination, growth rate & conidiation	Hypovirulent	Hypovirulence	[88]
	**METABOLISM**							
***mirC***: Siderophore iron transporter involved in synthesis of siderophores in intracellular microsomal compartment			***∆mirC*** (Iron-deplete condition)	Af ATCC 46,645	Reduce growth rate and conidiation	Normal	**/**	[66]
***Sid***: siderophore ***CpcA***: cross pathway control (transcriptional acgtivator) ***Paba***: gene encodes para aminobenzoic acid synthetase PABA, in folate biosynthesis pathway			***∆sidA***	Af ATCC 46,645	/	Avirulent	Avirulent	[58]
			***∆sidC***	Af ATCC 46,645		Hypovirulent	Hypovirulent	
			***∆sidF***	Af ATCC 46,645		Avirulent	Hypovirulent	
			***∆sidD***	Af ATCC 46,645		Hypovirulent	Avirulent	
			***cpcA***	Af D141		Avirulent	Hypovirulent	
			***paba***	Af 237		Avirulent	Avirulent	
***ArgEF***: encodes for 3 enzymes acetylglutamate synthase & kinase, acetylglutamyl-phosphate reductase ***ArgB***: encodes for ornithine transcarbamoyl transferase			***∆argEF***	*∆akuB* derivated from Af CEA17	Reduce growth rate & iron metabolism	Hypovirulent	**/**	[112]
			***∆argB***	Af293	No difference in growth & iron metabolism	Normal	/	
***ArgJ***: the only arginine biosynthetic enzyme lacking mammalian homologs			***∆argJ***	AfS77 (ATCC46,645)	/	Hypovirulent	Hypovirulent	[113]
***AmcA:*** mitochondrial transporter			***∆amcA*** (Iron-deplete condition + Nitrogen source)	Af S77 (ATCC 46645 *∆KuA*)	Reduce conidiation, growth rate & iron metabolism in presence of Glutamate or Ornithine	Normal	**/**	[60]
					No difference in fungal development & slight reduction of iron metabolism in presence of Arginine or Arginine + Ornithine		/	
***AcuM***: zinc cluster transcription factor			***∆acuM*** (Iron deficiency condition)	Af293	Reduced growth rate and iron metabolism	Hypovirulent	Hypovirulent	[65]
***LeuB***: transcription factor			***∆leuB*** (Normal or Iron deficiency condition)	Af A1160 ΔKu80 pyrG	Reduce growth rate in normal condition	Hypovirulent	**/**	[59]
					Increase or reduce growth rate in iron deficiency condition		/	
***PptA***: a phosphopantetheinyl transferase (P-pant)			***∆pptA***	Af A1160 ΔKu80 pyrG^+^	No production of secondary metabolites	Avirulent	Avirulent	[69]
***PcaA***: PIB-type cation ATPase which links metal homeostasis and heavy metal tolerance			***∆pcaA***	Af293	Reduce growth rate in presence of cadmium sulfate	Hypovirulent	**/**	[67]
					No difference in growth rate in presence of copper, iron, silver & zinc sulfate			
			***OEpcaA***	Af293	Increase growth rate in presence of cadmium sulfate	Normal	/	
					No difference in growth rate in presence of copper, iron, silver & zinc sulfate			
**Siroheme**: heme-like group used for sulfate and nitrate assimilation; ***met8:*** gene encoding the bifunctional dehydrogenase/ferrochelatase enzyme Met8			***∆met8***	AfS77	Role in growth rate & stress response	Hypovirulent	**/**	[61]

The analysis of the effect of virulence factors is based on the comparison of virulence of a wild strain with that of mutants obtained by gene deletion.

Increased: increased pathogenicity relative to wild strain; Decreased: decreased pathogenicity relative to wild strain;

No change: no change in the pathogenicity relative to wild strain; Avirulent: no pathogenicity with the mutan

#### Conidiation and germination

In fungi, six regulators of G protein signaling (RGS) domain proteins (flbA, gprK, rgsA, rax1, rgsC, and rgsD) are involved in fungal growth, sporulation, stress response, secondary metabolites, and virulence. Some of them negatively or positively regulate asexual development, gliotoxin or melanin production, and virulence of *A. fumigatus* in *G. mellonella* (Table 3). Thus, the Δ*rgsD* mutant displayed increased conidiation and elevated virulence [101], while the Δ*rgsC* [75] and Δ*gprK* [76] mutants showed reduced conidiation and increased germination, and decreased virulence in the larvae.

Other proteins are involved in cytoskeletal dynamics of *A. fumigatus*, as myosin (actin-based motor proteins family) that seems to have an important role in regulating virulence of *A. fumigatus* (Table 3). The *∆myoE* and *∆myoB* mutant strains had distinct effect on fungal development (delayed germination and reduced or increased conidiation, respectively) but were both hypovirulent in *G. mellonella* larvae [85].

#### Fungal growth

The calcium-calcineurin signaling pathway has an important role in fungal physiological processes, stress responses, and virulence [70, 86,87]

The Rab (Ras-related in brain) family of small GTPases (srgA A, srgA B, srgA C) were evaluated in *G. mellonella* model and showed their involvement in fungal development and filamentation. Only the ∆*srgA C* strain showed lower fungal virulence in *G. mellonella* larvae [89].

#### Septation

Septins, a conserved family of GTPases, are involved in a variety of critical cellular functions, including cell wall integrity and septation in *A. nidulans* [90,91]. On the other hand, *A. fumigatus* has five septins (aspA, aspB, aspC, aspD, and aspE) that seem necessary for septation but not for fungal growth [92] (Table 3). The ∆*aspA*, ∆*aspB*, and ∆*aspC* mutant strains were hypervirulent in *G. mellonella*. The virulence of ∆*aspB* strain was similar to that of the wild type strain in murine model [92].

### Secondary metabolites

*A. fumigatus* produces a wide range of secondary metabolites that can be harmful or beneficial. These small molecules of low molecular weight often have complex biosynthesis. Thus, Non-Ribosomal Peptide Synthetases (NRPS), key-enzymes involved in the biosynthesis of secondary metabolites in fungi [93], have many metabolic functions not yet elucidated. These secondary metabolites are necessary components since they enable the fungus to adapt itself to the host and grow inside it by escaping the immune response mechanisms. Other functions of these secondary metabolites are to facilitate tissue colonization and help the fungus tolerate external aggressions such as UV, desiccation, or competition with other micro-organisms [94]. Sequencing of the *A. fumigatus* genome showed the presence of 14 genes encoding for NRPS. *G. mellonella* model allowed researchers to study some NRPS functions, including gliotoxin production, as well as other molecules involved in acquisition of nutrients essential for fungal survival, such as iron (siderophores) (Table 3).

#### Secondary metabolites interacting with the immune response

Gliotoxin is best known secondary metabolites of *A. fumigatus*. It is a virulence factor which inhibits macrophage phagocytosis and oxidative response to stress, decreases cytotoxic activity of T cells, and hinders induction of apoptosis of host cells [95]. Of note, *G. mellonella* larvae mortality with the Δ*mtfA* strain is reduced [96] (Table 3). The mtfA transcription factor acts to regulate gliotoxin biosynthesis (via *gliZ* and *gliP* genes), in addition to its involvement in fungal growth and conidiation. On the same line, Reeves *et al*. showed a positive correlation between gliotoxin production and pathogenicity of selected *A. fumigatus* strains [48] originally differed in gliotoxin production. High rate of gliotoxin production by ATCC26933 strain was associated with high mortality in larvae, whereas ATCC16424, ATCC13073, and ATCC14109, the lower production of gliotoxin and caused less mortality in larvae (Table 3).

Melanin is another secondary metabolite and virulence factor of *A. fumigatus*. Melanin is a polymer of dihydroxynaphthalene (DHN) present on the surface of conidia to provide protection against UV and desiccation, in addition to its capacity to neutralize free radicals. Melanin-deficient mutants caused an increase of virulence in *G. mellonella* [97] (Table 3). Perhaps the absence of melanin could lead to a modification of the fungal cell wall which in turn triggered a greater immune response in the larvae.

Fumagillin, among the other mycotoxins of *A. fumigatus* analyzed in *G. mellonella* as a virulence factor, inhibits the action of neutrophils, a central element of the immune response to microbial infections. Fumagillin is produced during the development of *A. fumigatus* hyphae. A study reported that fumagillin inhibited the phagocytosis function of hemocytes, thus facilitating the growth of the fungus in the larva [47]. Therefore, pre-administration of fumagillin to larvae would increase susceptibility to *A. fumigatus* infection [98,99] (Table 3).

The ergot alkaloids are other metabolites produced by *A. fumigatus* (Table 3). The role of these alkaloids in the pathogenicity of *A. fumigatus* has been well studied *in vivo* in *G. mellonella. A. fumigatus* strains with ergot alkaloids mutations (fumigaclavine C deficiency) showed a virulence decrease. Fumigaclavine C is an inhibitor of TNF-alpha in human macrophages and could decrease expression of inflammatory cytokines in mice. PesL and pes1, involved in the final step of fumigaclavine C biosynthesis, have a role in the pathogenicity of *A. fumigatus* since Δ*pesL* was hypovirulent in *G. mellonella* [102]. O ’Hanlon *et al*. [102] found no difference in mortality compared with the reference strain ATCC46645, whereas Reeves *et al*. [103] observed a decrease in virulence upon using the wild-type strain Af293.1. Another gene, *dmaW*, implicated in the biosynthesis of fumigaclavine C, also had an effect on virulence of *A. fumigatus* in *G. mellonella* [104]. The mutant Δ*dmaW* inhibited the synthesis of final product fumigaclavine C, and consequently lowered the virulence of *A. fumigatus* in *G. mellonella*.

#### Secondary metabolites of A. fumigatus involved in iron metabolism

**Figure 4.**
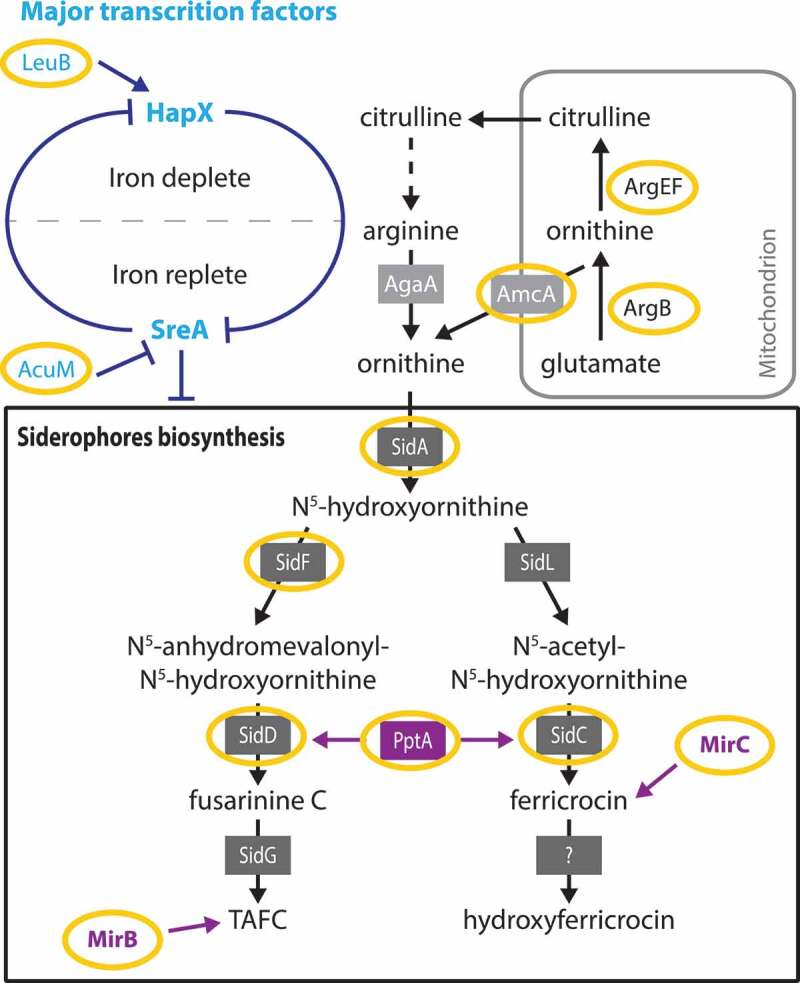
Iron metabolism of *A. fumigatus* studied in *G. mellonella* (adapted from [109])

Two types of siderophores are described in *A. fumigatus*: extracellular hypha-secreted siderophores [fusarinin C (FSC) and triacetylfusarinin C (TAFC)], and intracellular siderophores for iron storage and distribution in hyphae (Ferricrocin (FC)) or in conidia (hydroxyferricrocin (HFC)). The first stage of siderophores biosynthesis consists in hydroxylation of ornithine catalyzed by SidA. Schrettl *et al*. showed that *∆sidA* led to avirulence of the strain in mice [105,106] while Slater *et al*. found concordant results in *G. mellonella*, regardless of the mutant inoculated dose [107]. Other genes implicated in both pathways of siderophores biosynthesis, like *sidC* (intracellular siderophore) and *sidD* or *sidF* (extracellular siderophores), have also been tested in rodent or *G. mellonella* models (Figure 4). The *∆sidF, ∆sidD*, and *∆sidC* mutants induce reduced virulence in mice [106] and in *G. mellonella* model [107] (Figure 4). In mice as in *G. mellonella* model, deletion of genes coding for the first steps of the siderophore biosynthesis pathway could have a big effect on the virulence of *A. fumigatus*. However, deleting genes encoding for late-stage mechanisms had no such effect due to the presence of alternative pathways (Table 3).

Yellow circles: steps of iron metabolism of *A. fumigatus* studied in *G. mellonella*

Johns *et al*. showed that PptA, a putative 4ʹ-phosphopantetheinyl transferase (4ʹ-PPTase), has a non-redundant role in the production of different secondary metabolites, like gliotoxin, DHN-melanin, and siderophores (TAFC and FC) [108]. The PptA null mutant *(∆PptA*) is avirulent in *G. mellonella* larvae and in both bronchopulmonary and disseminated murine infection models (Figure 4).

In fungi, siderophores are absorbed by siderophore-specific transmembrane transporters, siderophore iron transporter (SIT), a subgroup within the major facilitating superfamily (MFS) [109]. Of those SITs, two have been distinguished for their role in mediating TAFC uptake (MirB) or in intracellular siderophore biosynthesis (MirC) (Figure 4) [110]. When Δ*mirC* mutant was inoculated in *G. mellonella* in an iron-poor environment, production of ferricrocin (intracellular siderophore) and virulence decreased [111]. These results confirm the involvement of MirC in the regulation of iron metabolism and its implication in the pathogenicity in *G. mellonella*.

Although most of the key steps of siderophore biosynthesis were studied in *G. mellonella* model (Figure 4), highlighting its importance and its implication in the fungal pathogenicity, many of this metal transporters in *A. fumigatus* have not been characterized yet. Other genes involved in the homeostasis of nutrients have been studied and shown their importance in *A. fumigatus* virulence in *G. mellonella* model (Table 3) [112].

## Comparison between *G. mellonella* and murine models

To validate the *G. mellonella* model, several authors compared the results on *G. mellonella* with those on the murine model, with, in most cases, a good correlation. For fungal analysis, studies of virulence factors of *Mucor circinelloides* [71], *Fusarium species* [56], and *Candida albicans* [113] were compared in both *G. mellonella* and mice, showing that genes activated to yield full virulence in larvae and in immunodepleted mice were the same. The results are comparable. On the other hand, Amorim-Vaz *et al*. examined transcription factors involved in virulence of *C. albicans* by comparison of the two models [20]. They considered *G. mellonella* as a useful model even though there was only 50% concordance between results in mice and *G. mellonella* larvae. Interestingly, another publication found discrepancy of pathogenicity of strains of *C. albicans* [103]. In our review and by analyzing mutants from *A. fumigatus* in *G. mellonella*, it is important to note that in consistency with Amorim-Vaz *et al*. about half of the comparisons showed good correlation. All these results support the presence of similitudes in the mechanisms of fungal infection between the rodent and *G. mellonella* models, but the discrepancies suggest that the lack of adaptive immune system in the larvae disrupts the perfect alignment between the two model types. Although most data are well correlated between the two models, in mammals, results can be different because of the interaction with a more complex immune system than in insect.

## Conclusion

Larvae of *G. mellonella* present several interesting criteria that encourage researchers to use it as an *in vivo* model, hence the increased number of publications on molecules or pathogens that have been tested on larvae in recent years. The possibility of conducting large-scale studies using this mini-host model makes it a powerful tool; however, many teams have noticed that different outstanding parameters may modify the larval immune response and thus influence the results of experimental infection. It is important to remedy these issues with standardization of study design, which has started to develop recently. Additionally, complete sequencing of the genome will open the door wide for further research using this model.

Thanks to similarities between mammal and insect innate immune systems, *G mellonella* could be used to understand infection mechanisms and to assess virulence of different pathogens, including fungi, especially *A. fumigatus*. The latter is one of the most pathogenic fungi against which researchers endeavor to identify new therapeutic targets, as this is becoming one of the public health issues of particular concern. Studying *A. fumigatus* isolates’ pathogenicity is necessary by analyzing the production of their arsenal of secondary metabolites or say virulence factors via the strategy of gene disruption. In the last 5 years, several studies have explored the impact of metals like iron, and the production of mycotoxin or proteins on their virulence in *G. mellonella* model. From this review, it appears that *A. fumigatus* can produce a vast array of active biomolecules and virulence factors that could enhance its pathogenicity. Some signaling pathways were almost entirely studied in the larvae, which proves the high interest of utilizing them to initiate large-scale pre-screening protocols, conducted in mammals, for the identification of potential therapeutic drugs, in compliance with the 3Rs.

To conclude, the *G. mellonella* model, by all its advantageous characteristics, proven its utility to study host-pathogen interactions, particularly for *A. fumigatus*. It can serve as a fast, simple, and low-cost pre-screening model to complete data before using a mammalian model, in a medical field where a great part of progress is necessary to optimize patient management.
